# Designing Antibacterial Peptides with Enhanced Killing Kinetics

**DOI:** 10.3389/fmicb.2018.00325

**Published:** 2018-02-23

**Authors:** Faiza H. Waghu, Shaini Joseph, Sanket Ghawali, Elvis A. Martis, Taruna Madan, Kareenhalli V. Venkatesh, Susan Idicula-Thomas

**Affiliations:** ^1^Biomedical Informatics Centre, ICMR-National Institute for Research in Reproductive Health, Mumbai, India; ^2^Molecular Simulation Group, Department of Pharmaceutical Chemistry, Bombay College of Pharmacy, Mumbai, India; ^3^Department of Innate Immunity, ICMR-National Institute for Research in Reproductive Health, Mumbai, India; ^4^Department of Chemical Engineering, IIT Bombay, Mumbai, India

**Keywords:** antibacterial peptides, MD simulation, killing kinetics, microbial membrane, rational design

## Abstract

Antimicrobial peptides (AMPs) are gaining attention as substitutes for antibiotics in order to combat the risk posed by multi-drug resistant pathogens. Several research groups are engaged in design of potent anti-infective agents using natural AMPs as templates. In this study, a library of peptides with high sequence similarity to Myeloid Antimicrobial Peptide (MAP) family were screened using popular online prediction algorithms. These peptide variants were designed in a manner to retain the conserved residues within the MAP family. The prediction algorithms were found to effectively classify peptides based on their antimicrobial nature. In order to improve the activity of the identified peptides, molecular dynamics (MD) simulations, using bilayer and micellar systems could be used to design and predict effect of residue substitution on membranes of microbial and mammalian cells. The inference from MD simulation studies well corroborated with the wet-lab observations indicating that MD-guided rational design could lead to discovery of potent AMPs. The effect of the residue substitution on membrane activity was studied in greater detail using killing kinetic analysis. Killing kinetics studies on Gram-positive, negative and human erythrocytes indicated that a single residue change has a drastic effect on the potency of AMPs. An interesting outcome was a switch from monophasic to biphasic death rate constant of *Staphylococcus aureus* due to a single residue mutation in the peptide.

## Introduction

Antimicrobial peptides (AMPs) are diverse, biologically active molecules of the innate immune system. AMPs exhibit broad spectrum antimicrobial activity. AMPs have multiple cellular targets (Lohner, 2017) and this makes it difficult for microbes to develop resistance against them as compared to antibiotics which mostly have a single target. Furthermore, they also exert several immunomodulatory properties such as modulation of cytokine and chemokine expression, leukocyte activation (Sorensen et al., 2008) etc. Studies have shown that dysregulation of the generation of AMPs in innate immune responses results in increased susceptibility to microbial infections (Ganz, 2003; Porto et al., 2016; Modi et al., 2017). AMPs also play a role in other biological processes such as angiogenesis and wound healing (Veldhuizen et al., 2014).

Due to their broad-spectrum activity and limited scope for developing resistance, AMPs are widely studied as substitutes to conventional antibiotics that are fast becoming ineffective against many microbes (Chung and Khanum, 2017). Presently, we have only a handful of AMPs that have been tested in phase III trials for their therapeutic use. Few of the reasons for their failure are *in vivo* stability, efficiency and toxicity due to which AMPs have been discontinued from the undergoing clinical trials (Mahlapuu et al., 2016).

Responding to this need, there has been a surge in research efforts focused on rational design and prediction of AMPs (Fjell et al., 2011; Porto et al., 2017a). Several studies have employed rational design strategies to generate novel AMPs with improved activity and reduced toxicity. The *de novo* rational design method involves identification of sequence patterns, crucial residue positions and amino acid frequencies from known AMPs. This information is then used to develop prediction method and linguistic models to identify novel AMPs. Another approach used to rationally design novel AMPs is the template based method where substitutions/truncations in the native peptide may lead to design of more potent AMPs with reduced toxicity. This exercise is therefore strongly dependent on highly sensitive algorithms that can accurately predict the change in membrane activity due to minor sequence variations (Porto et al., 2012).

In this study, we used template-based approach to create a library of peptides that share high sequence similarity to MAP antimicrobial peptide family and these were screened using online prediction algorithms. Three of the predicted peptides were tested for their antibacterial activity using wet-lab methods. We further aimed to increase the membrane activity of one of the identified novel AMPs by MD-guided rational design. The increase in potency brought about by the predicted single residue substitution was studied using Gram-positive and negative strains of bacteria as well as human erythrocytes. The microbial death by peptides is characterized by minimum inhibitory concentration (MIC) value. MIC can be determined by fitting Hills Equation to data representing percentage death obtained in a specific time and peptide concentration. Hills equation is represented by two parameters, Hills coefficient, which determines the sensitivity of response and, half saturation constant, which represents the threshold concentration needed for 50% death (Yu et al., 2016). The efficacy of a peptide will be determined by a high value of Hills coefficient (ultrasensitive) and a low half saturation constant (lesser MIC-value). In this case, first order kinetic constant for death at a peptide concentration is evaluated to determine the efficacy of a peptide. The rate constant is a function of the peptide concentration and increases with increase in the peptide concentration (Verma et al., 2003; Regoes et al., 2004).

## Materials and methods

### Peptide design

Myeloid antimicrobial peptides exhibit potent broad spectrum antibacterial, antifungal, anticancer, and antiviral activity. Thus, sequence information of this family was used for design of novel membrane active AMPs. Residues conserved within the family were identified and an in-house perl script was used to virtually generate 1,000 random peptide sequences by replacing the non-conserved residues within the family. These sequences were then run through the CD-HIT webserver (Huang et al., 2010) to retain a dataset of 935 sequences with <85% identity (Supplementary Table 5). Popular, online algorithms viz., AntiBp2 (Lata et al., 2010), ADAM (Lee et al., 2015) and CAMP_R3_ (Waghu et al., 2016), iAMP-2L (Xiao et al., 2013) were used to predict the antimicrobial activity of these peptides. Seven of the 935 peptides had prediction scores greater than that of the known antimicrobial peptide BMAP-28(1–18). Three sequences (P1, P2, and P3) were randomly selected from these seven for further experimental validation.

### Peptide synthesis

The peptides [BMAP28(1–18), P1, P2, P3, and P1m] with N- terminal amino-lauric acid and C-terminal amide conjugation were synthesized commercially (Peptide2, USA) using solid phase F-moc chemistry and purified by reverse-phase high performance liquid chromatography to a purity of >95%. The N & C-terminal modifications were carried to improve the activity and stability of the peptides (Brinckerhoff et al., 1999; Rotem et al., 2006).

### Antimicrobial assay

Antimicrobial activity of peptides against Gram-positive (*Staphylococcus aureus ATCC 25923, S. aureus ATCC 6538P*) and Gram-negative (*Escherichia coli ATCC 25922, E. coli ATCC 8739*) bacteria were determined by microdilution method. Bacterial cultures were grown in Mueller–Hinton (MH) broth at 37°C for 18 h on a shaker to obtain an OD600 nm of 0.8. The cultures were diluted in MH broth to obtain a cell density of 10^5^-10^6^ c.f.u/ml. The peptides were serially diluted in MH broth. The serially diluted peptides were inoculated with bacterial cultures in 1:1 ratio in sterile 96-well microtiter plates. A bovine myeloid antimicrobial peptide, BMAP28(1–18), was used as the positive control. The peptides were tested for antimicrobial activity in the range from 1 to 100 μM. The MICs of the peptides were determined by OD600 nm measurements after 18 h of incubation at 37°C. Bacterial strains were purchased from National Collection of Industrial Microorganisms, National Chemical Laboratories, Pune, India. The percentage death evaluated for *S. aureus ATCC 25923* and *E. coli ATCC 8739* after 18 h incubation was fitted to a Hill equation and the parameters namely Hill coefficient (n) and the half-saturation constant (K) were evaluated (Details in Supplementary File).

### Molecular dynamics simulation

#### Generation of helical conformation of the peptides

BMAP28(1–18) adopted helical conformation in SDS (Skerlavaj et al., 1996). Therefore, the designed peptides were modeled in helical conformation and energy minimized using 200 steps of steepest descent algorithm in vacuum using Discovery studio v3.5 (Accelrys, San Diego, CA).

#### Preparation of peptide lipid systems

All peptide-lipid systems were created using CHARMM-GUI input generator (Wu et al., 2014; Lee et al., 2016; Jo et al., 2017). The details of individual systems are depicted in (Supplementary Table 1).

#### Preparation of BMAP28(1–18) in lipid bilayer

The bilayer was solvated using TIP3P water models (Jorgensen et al., 1983) to attain a solvation shell of 20 Å on either side of the bilayer. The protein was initially placed at a distance of ~40 Å from the center of mass (COM) of the bilayer to allow unbiased peptide-lipid contact. K^+^ and Cl^−^ ions were added to maintain a physiological salt concentration of 0.15 M.

#### Preparation of P1, P1m, P1m1, and P1m2 in SDS micelle

The SDS micellar system that was built using CHARMM-GUI (Cheng et al., 2013) was initially minimized using steepest descent algorithm, followed by 500 ps of equilibration under NVT ensemble and another 500 ps of equilibration under NPT ensemble. Each peptide was placed in the equilibrated simulation box with its COM coinciding with that of the micelle. The peptides were placed along the micelle diameter. The peptides were neutralized by adding Cl^−^ ions in the aqueous phase.

#### Preparation of P1 and P1m in DPC micelle

The peptides were placed with their COM coinciding with that of the dodecylphosphocholine (DPC) micelle that was built using CHARMM-GUI. K^+^ and Cl^−^ ions were added to maintain a physiological salt concentration of 0.15 M.

#### Molecular dynamics simulations

All MD simulations were performed using Gromacs 4.6.5 (Pronk et al., 2013) with CHARMM force field (Brooks et al., 2009). The details of the parameters used for the simulations can be seen in Supplementary Table 1. During all phases of the simulations, including the equilibration phases, the long range electrostatic interactions were treated using the fast smooth particle mesh Ewald summation (PME) method. The Verlet cut-off scheme was used to treat the short range pairwise interactions that was truncated beyond 12 Å and this list was updated every 20 steps. All the production phase simulations employed the Nose-Hoover temperature coupling method and the Parrinello–Rahman method for pressure coupling. The leap-frog algorithm (Gunsteren and Berendsen, 1988) was used to integrate the Newton's laws of motion.

#### Equilibration and production phase for BMAP28(1–18) in lipid bilayer

This system was thoroughly minimized using steepest descent algorithm for 100,000 steps. It was heated to 303.15 K under the NVT ensemble for 1 ns with the integration time-step of 1 fs, followed by an additional step of 1 ns equilibration run under NVT ensemble (Bruce et al., 2002; Lyu et al., 2015; Pino-Angeles et al., 2016; Xiang et al., 2016). The density equilibration was performed under NPT ensemble for three steps of 1 ns each using 2 fs as the integration time step that was achieved by switching on the LINCS algorithm to constrain all bonds involving hydrogen atoms. The system was coupled to an external temperature and pressure bath using the Berendsen's thermostat and barostat, respectively. A semi-isotropic scheme for pressure coupling was used along the Z-axis. The production phase of the simulation lasted for 100 ns. The conformations were saved every 10 ps for analysis.

#### Equilibration and production phases for peptides in SDS and DPC micelle

The system, under weak restraints, was minimized using the steepest descent algorithm for 55,000 steps. The restraints were gradually removed in 25,000 steps and the entire system was further minimized for 20,000 steps without any restraints. The system was heated to 300 K under NVT ensemble for 500 ps, followed by 500 ps of density equilibration under the NPT ensemble.

#### Analysis of MD simulations

The trajectories obtained from the production run of MD simulations were further analyzed using Gromacs tools. The radial distribution function (RDF), root-mean-squared deviation (excluding waters) and moment of inertia were analyzed using *g_rdf, g_rms*, and *g_gyrate* functions, respectively. Eccentricity (*e*) was calculated as:

$$\text{e}=1-\frac{I_{\text{min}}}{I_{\text{avg}}}$$

where *I*_min_ is the moment of inertia along the x, y, or z axis with the smallest magnitude and *I*_avg_ is the average of all three moments of inertia (Bruce et al., 2002).

### Circular dichroism (CD) spectroscopy

CD measurements of the peptides [BMAP28(1–18), P1, and P1m] were performed on a Jasco J-815 spectropolarimeter (JASCO, Japan) at 298 K. CD spectra were recorded in continuous scan mode using a rectangular cuvette of 1 mm path length and a bandwidth of 1 nm for wavelength ranging from 190 to 260 nm. Each spectrum was an average of 3 scans. Lyophilized peptides having concentration of 100 μM were dissolved in 10 mM phosphate buffer (pH 7.4). CD was carried out in (a) phosphate buffer and (b) in the presence of 25 mM sodium dodecylsulfate (SDS). All spectra were baseline corrected by subtraction of the CD spectra of the phosphate buffer and were smoothened to make sure that the overall shape of the spectra was not altered.

### Cytotoxicity assay against human erythrocytes

Hemolysis was determined by incubating 10% (v/v) suspension of human RBCs in PBS (pH 7.4) with different concentrations of peptides [BMAP28(1–18), P1, and P1m] ranging from 1.56 to 100 μM at 37°C for 1 h. RBCs incubated with 0.1% Triton X-100 was used as the positive control and RBCs incubated with PBS was used as blank. The incubated mixtures were centrifuged at 500 × g for 5 min. The hemolytic activity of the peptides was determined by measuring the absorbance of the supernatant at 541 nm. The percent hemolysis was calculated using the equation:

$$\text{H}=\left(\frac{absorbance\;of\;sample-absorbance\;of\;blank}{absorbance\;of\;positive\;control-absorbance\;of\;blank}\right)\times 100$$

The percentage hemolysis (H) was fitted using a Hill function with respect to the peptide concentration.

### Killing kinetics assay

Overnight grown culture (*S. aureus* ATCC 25923) was diluted in fresh MH broth and allowed to grow to obtain OD600 nm of 0.8. The culture was diluted in MH broth to obtain a cell density of 10^5^-10^6^ c.f.u/ml and was incubated with the peptides at various concentrations within the MIC range for each peptide [BMAP28(1–18), P1, and P1m] till a highest concentration of 50 μM. Aliquots were drawn at specific time intervals and plated after dilution on MH agar plates. The plates were incubated at 37°C for 24 h to obtain viable colonies for calculating the initial death rates of the peptides. The kinetic rate constant was determined by evaluation of c.f.u count at various time points ranging from 10 to 45 min. First order death rate constant was determined by plotting log(c.f.u) vs. time and the slope of linear fit yielded the death rate constant. Four to five time points were used to determine the rate constant. Only two time points were considered for death rate constant at very high concentration (50 μM) as complete killing occurred in the first 15 min.

## Results and discussion

### Antimicrobial activity of the designed peptides

Three novel antibacterial peptides (P1, P2, and P3) that shared high sequence similarity (see section Materials and Methods) were predicted using online algorithms (Table 1). The designed peptides were synthesized and assayed for antibacterial activity against Gram-positive (*S. aureus*) and Gram-negative (*E. coli*) bacterial strains. A known myeloid antimicrobial peptide, BMAP28(1–18), was used as the positive control. The designed peptides share equal length and 55% sequence identity with BMAP28(1–18). Two of the three designed peptides were found to be antibacterial (Table 2).

**Table 1:** Sequence information of the designed peptides.

Peptide^*^	**Sequence^#^**	**Identity to CAMP's AMPs (%)**	**CAMP accession**
P1	VL**LR**A**L**A**RKI**TLGI**KKYG**	69	CAMPST23 (SMAP29)
P2	CI**LR**W**L**A**RKI**PWHA**KKYG**	63	CAMPST23 (SMAP29)
P3	VF**LR**I**L**V**RKI**APGV**KKYG**	81	CAMPST23 (SMAP29)

# *Identical residues amongst the 3 peptides are written in bold*.

* *All the peptides are predicted to be antimicrobial by CAMP_R3_, ANTIBP2, ADAM, and iAMP-2L algorithms*.

**Table 2:** MIC (μM) ^#^ of peptides.

**Peptides**	***S. aureus* 6538 P**	***S. aureus* 25923**	***E. coli* 8739**	***E. coli* 25922**
BMAP28 (1–18)	3.125–6.25	3.125–6.25	3.125–6.25	25–50
P1	6.25–12.5	6.25–12.5	50–100	50–100
P2	12.5–25^*^	50–100^*^	50–100^*^	NA
P3	50–100^*^	NA	NA	NA
P1m	3.125–6.25	3.125–6.25	6.25–12.5	25.50

# *Minimal inhibitory concentration (MIC) was the average range of values obtained from triplicates of three independent experiments*.

* *50% inhibition, NA-inactive upto 100μM*.

P1 was found to be the most potent amongst the designed peptides against the tested strains of bacteria. The order of antibacterial activity of the peptides determined experimentally was P1>P2>P3. Of the evaluated algorithms, the predictions of SVM and RF algorithms of CAMP seemed to be closest to the experimental observations. Recently, an attempt to evaluate the web-based antimicrobial prediction tools revealed that among the six general AMP prediction algorithms viz., ADAM (Porto et al., 2012), CAMP_R3_ RF & SVM (Waghu et al., 2016), MLAMP (Lin and Xu, 2016), DBAASP (Vishnepolesky and Pirtskhalava, 2014), and AMPA (Torrent et al., 2012); CAMP_R3_ RF demonstrated statistically significant improvement in performance, as determined by comparison of the ROC curves (Gabere and Noble, 2017).

Most of the existing sequence-based prediction algorithms rely on physicochemical properties of the constituent amino acids and hence may not capture impact of subtle changes in peptide composition brought about by conserved substitutions, shuffling (Porto et al., 2017b) etc. Hence these antimicrobial prediction servers may not aid in rational design efforts that aim to enhance the potency of existing antimicrobial peptides. An alternate, albeit resource intensive *in silico* approach for template/knowledge-based rational design is MD simulations. In order to explore the utility of this method, lipid bilayer MD simulation studies were performed on BMAP28(1–18) to delineate the critical residue/s for lipid interaction and subsequently use this information for improving the potency of P1.

### Identification of residue positions that strengthen peptide-membrane interaction

**Figure 1:**
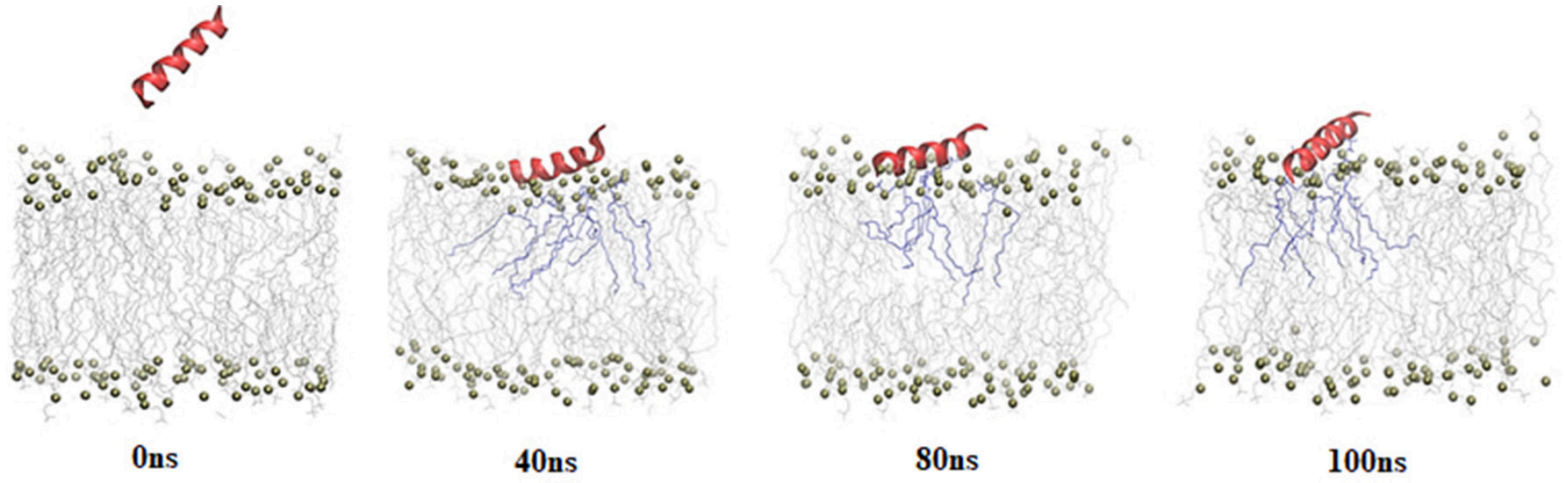
Snapshots of 100 ns simulation of BMAP28(1-18) with POPC: POPG bilayer. The peptide is represented in cartoon format; lipid head groups are shown as balls; tails are shown as lines and lipid tails within 3 Å of the peptide are highlighted in purple.

A 100 ns simulation of BMAP28(1–18) with 2:1 composition of POPC:POPG lipid bilayer model was carried out (Supplementary Figure 1). This lipid bilayer model mimics a bacterial membrane. BMAP28(1–18) was initially placed ~40 Å away from the COM of the bilayer in helical conformation to allow unbiased peptide lipid interaction. Representative snapshots from the 100 ns simulations of BMAP28(1–18) and the bilayer are shown in Figure 1.

Peptide-lipid bilayer interaction is mainly governed by electrostatic and hydrophobic forces. Peptide residues involved in interaction with lipid bilayer were identified by calculating the distance of COMs of each residue and the lipid head groups averaged over the entire simulation period. This distance was also computed for the cluster representative having highest cluster members (Supplementary Figure 2). It was observed that Arg8, Leu11, and Arg12 exhibited high proximity to the lipid bilayer with Leu11 being the nearest to the lipid groups (Supplementary Figure 2).

The strength of interaction between the charged and hydrophobic (based on positive Kyte and Doolittle score) residues of the peptide with the lipid head group was inferred based on the RDF plot. RDF-values are positively correlated to the strength of interaction with the lipid head groups. Arg4 and Leu11 exhibited high RDF-values and is therefore predicted to have strong interaction with the lipid head groups (Supplementary Figure 3).

Thus, distance and RDF calculations predicted the 11th position to be the most critical for strengthening peptide-lipid interaction and consequentially for antimicrobial activity.

Helical wheel analysis of the hydrophobic phase of P1 revealed that it contained two polar residues Thr11 and Tyr17 (Supplementary Figure 4). Hence, in order to validate the importance of the 11th position (identified by bilayer simulations), two *in silico* mutants Thr11Leu (P1m) and Tyr17Leu (P1m1) were designed and the effects of these substitutions on SDS micelle were studied by MD simulations. It is to be noted that Tyr17 is a conserved residue amongst the MAP family of proteins.

To understand the influence of hydrophobicity and/or volume at position 11, a third mutant of P1 (Thr11Val; P1m2) was designed.

### Effect of mutation on structure of SDS and DPC micelles

In order to predict the effect of mutations on antimicrobial activity, the mutants (P1m, P1m1, and P1m2) were subjected to MD simulation studies using SDS micelle which is a simple system that mimics the microbial membrane (Supplementary Figure 5).

**Figure 2:**
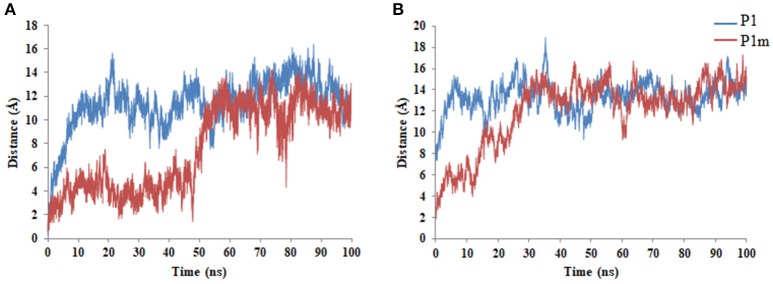
Time profile of the distance between the COMs of the peptides and **(A)** SDS **(B)** DPC during the full course of the simulation.

All peptides being amphipathic in nature diffuse from the center to the periphery of the micelle to achieve an energetically favorable conformation. P1m takes a much longer time to reach the periphery of SDS as compared to P1, P1m1, and P1m2. This may be attributed to stronger interaction of P1m with the micelle as compared to the other peptides. All peptides achieve equilibrium around 50 ns as can be seen from the distance profile between COMs of the peptides and the micelle (Figure 2A and Supplementary Figure 6).

Eccentricity (*e*) and moment of inertia (MOI) values were calculated to observe the effect of mutation on structure of SDS micelle during the stabilized period of simulation (50–100 ns). For a perfect sphere, *e*-value is 0 and R1:R2:R3 is 1:1:1 (Khandelia and Kaznessis, 2005; Saviello et al., 2010). Thus any effect on the structure can be determined by monitoring eccentricity and moment of inertia. SDS simulations with P1m and P1m2 exhibited more elevated *e*-value as compared to P1 and P1m1. P1m exhibited more elevated MOI-values as compared to P1, P1m1, and P1m2 indicating a stronger interaction of P1m with SDS leading to perturbation of its structure (Supplementary Table 2).

#### Calculation of relative binding free energy

The stabilized simulation period of 50–100 ns was used to calculate the relative binding free energy of the peptide-SDS complex. Antimicrobial activity is known to be positively correlated to favorable binding energy (Sayyed-Ahmad et al., 2009). *g_mmpbsa* tool (Kumari et al., 2014) which implements the MM-PBSA (Molecular mechanics Poisson-Boltzmann surface area) approach was used to compute the relative binding free energies of P1, P1m, P1m1, and P1m2 to SDS micelle. It was observed that P1m displayed more favorable relative binding free energy to SDS micelle as compared to P1, P1m1, and P1m2 (Supplementary Table 3).

Based on the analysis of the distance profiles (Supplementary Figure 6), e, MOI (Supplementary Table 2) and relative binding free energy (Supplementary Table 3), the order of antimicrobial activity was predicted as P1m(Thr11Leu)>P1m2(Thr11Val)>P1m1(Tyr17Leu)>P1. These results indicated (i) the importance of the 11th position for antimicrobial activity, (ii) position 11 is influenced by both hydrophobicity and volume and (iii) not all improvement/s in charged or hydrophobic face of helix may equally improve the MIC.

In order to predict the effect of Thr11Leu mutation on toxicity of the peptides, P1 and P1m were subjected to MD simulations using DPC micelle which is a simple system to mimic the mammalian membrane (Supplementary Figure 7). The peptides diffuse from the center to the periphery of the micelle to achieve an energetically favorable conformation as can be seen from the distance profile between COMs of the peptides and the micelle (Figure 2B). The Thr11Leu mutation in P1m enhanced the distortion of DPC micelle (Supplementary Table 2) and exhibited more favorable relative binding free energy as compared to P1 (Supplementary Table 4).

Thus, the Thr11Leu mutation seems to enhance membrane activity of P1m against both microbial and mammalian cells as inferred from the observations of SDS and DPC micelle simulations, respectively.

#### Effect of mutation on antibacterial and hemolytic activity of peptides

**Figure 3:**
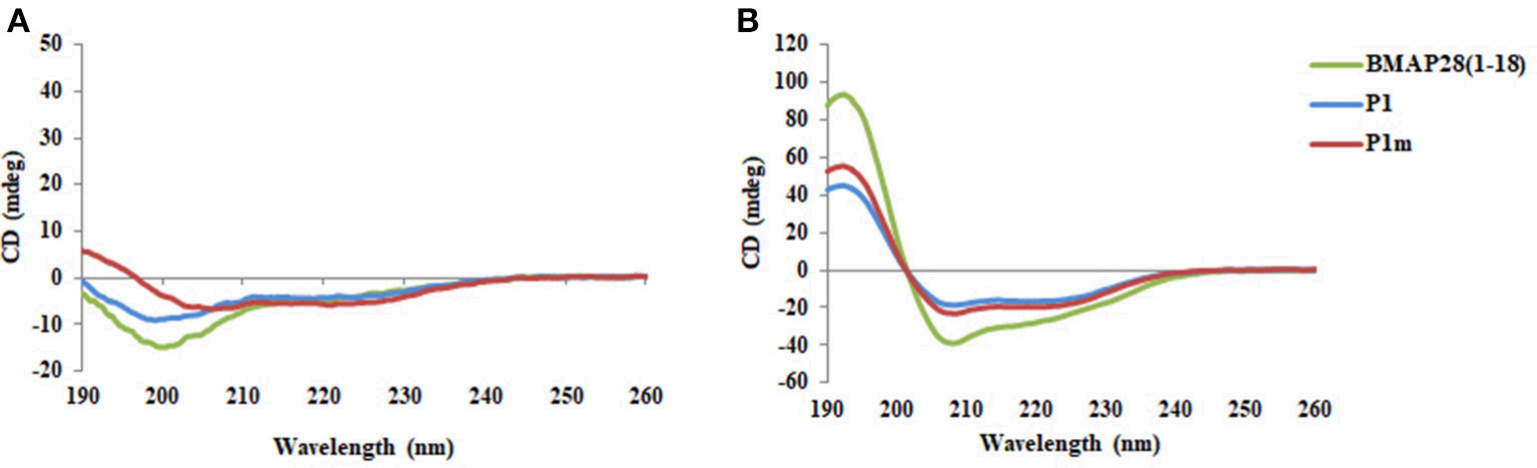
CD spectra of peptides BMAP28(1–18), P1, and P1m in the presence of **(A)** 10 mM PB and **(B)** 25 mM SDS.

In order to corroborate the *in silico* predictions, P1m was synthesized. BMAP28(1–18), P1, and P1m were found to adopt a helical conformation in SDS (Figure 3). The effect of mutation on membrane activity was studied in detail on three different membranes viz., Gram-positive, Gram-negative and human erythrocytes. The data was fitted to Hill equation which yields two parameters, Hill coefficient (*n*) and the half saturation constant (K). The Hill coefficient, *n*, denotes the sensitivity of the response, with *n* = 1 indicating a typical Michaelis-Menten response and a value of *n* > 1 indicating an ultra-sensitive response. Ultra-sensitivity arises due to multiple peptides binding or aggregation of several peptide molecules onto the cell membrane.

The half saturation constant represents the threshold level of peptide required for 50% death. A lower value of *K* indicates amplification in the response. *K* also represents the binding affinity of the peptide, quantitatively indicating the dissociation constant. Table 3 lists the Hill parameters (*n* and *K*) for Gram-positive, negative and human RBCs.

**Table 3:** Calculated Hill parameters for three different membranes.

**Peptides/Membrane**	**Gram** +**ve (*****S. aureus*** **25923)**		**Gram** −**ve (*****E. coli*** **8739)**		**Human RBCs**	
	n^*^	**K^#^**	n^*^	**K^#^**	n^*^	**K^#^**
BMAP28(1–18)	2.5	1.4	3.07	2.2	1.1	61.4
P1	1.2	3	1.02	26.6	1.55	170
P1m	4.5	2.2	2.13	3.8	1.3	14.2

* Hill coefficient;

# *half saturation constant*.

#### Case 1: gram-positive bacteria

**Figure 4:**
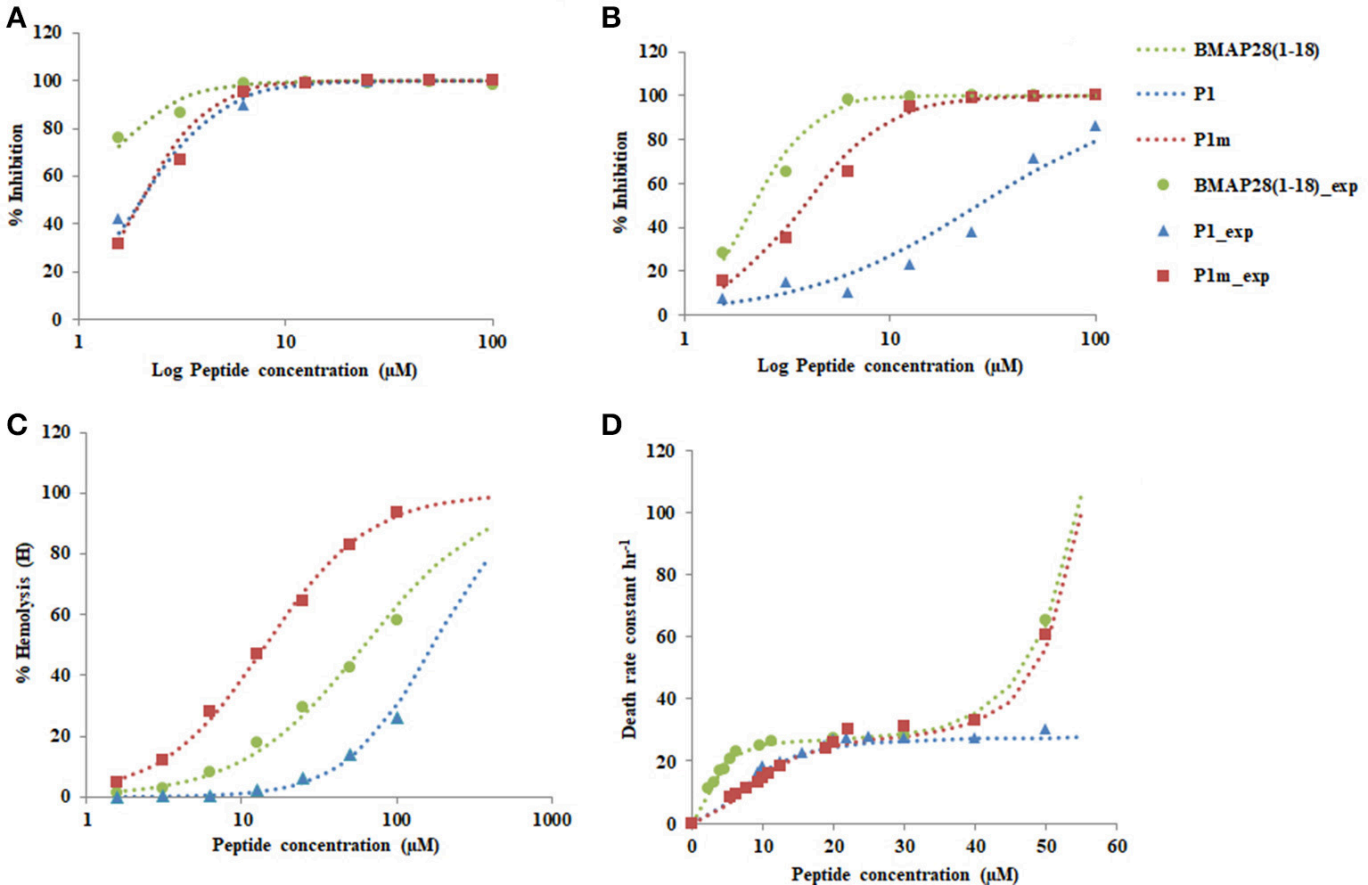
Plot of % Inhibition of **(A)** *S. aureus* 25923 **(B)** *E. coli* 8739 **(C)** % Hemolysis **(D)** Death rate constant vs. the concentration of the peptide. Dotted lines represent the fitted values and symbols represent the observed values of the experiment.

The single residue mutation significantly enhanced the antibacterial activity of the peptide (Figure 4A). The Hill coefficient (*n*) was highest for P1m indicating a highly sensitive killing response followed by BMAP28(1–18) and then by P1. The half saturation constant (*K*) for the peptides were of similar magnitude (Table 3).

To further study the effect of mutation on behavior of peptides, the first order rate constant for the death of *S. aureus* was determined by performing time-kill assays using BMAP28(1–18), P1, and P1m. The death rate constant as a function of peptide concentration yielded an interesting behavior which has not been reported previously. The kinetic constant demonstrated biphasic behavior for BMAP28(1–18) and P1m and a monophasic behavior for P1(Figure 4D).

The biphasic behavior observed for BMAP28(1–18) and P1m demonstrated two distinct phases, an initial saturating phase (fitted by Hill equation) where the killing rate saturates and a second phase of rapid killing with an exponential increase in the death rate constant. The first phase appears to represent a binding mechanism of peptide to the membrane, whereas the second may represent lysis captured by increased death rate. BMAP28(1–18) demonstrated a higher affinity at lower concentrations than that for P1m, but demonstrated a similar exponential increase beyond the saturating peptide concentration. P1, however, showed a lower affinity at lower concentration similar to that of P1m, but indicated saturation and a possible delayed exponential phase. Thus, P1m behaves similar to P1 at lower concentrations and alters its behavior to mimic BMAP28(1–18) at higher concentrations. It is interesting to note that the exponential behavior was quenched due to a change in single residue in the range of peptide concentrations studied. However, the underlying mechanism linking the sequence and the biphasic behavior is unclear. We speculate that this may be dependent on the peptide binding characteristics to the cell membrane and the actual lysis of the cell.

#### Case 2: gram-negative bacteria

In case of Gram-negative bacteria, *E. coli*, BMAP28(1–18) and P1m demonstrated ultrasensitive response with a high *n*-value, while P1 exhibited a Michaelis-Menten type response (Table 3). Further, the half saturation constants indicate that both in case of BMAP28(1–8) and P1m, the responses were amplified by 14- and 7-fold, respectively relative to P1. Thus, both BMAP28(1–18) and P1m demonstrated a higher antimicrobial activity in case of Gram-negative bacteria relative to P1 (Figure 4B).

#### Case 3: human erythrocytes (RBCs)

The peptides were tested for their ability to hemolyse human erythrocytes. All peptides were found to be hemolytic (Figure 4C). The Hill coefficients were of similar magnitude for the three peptides (Table 3). However the half saturation constant altered dramatically for BMAP28(1–18), P1, and P1m.

The reduction of about 12-fold in the half saturation constant for P1 to P1m indicates amplification of hemolysis due to a single residue change. The half saturation constant for P1m was also about 4.3-fold lower from that observed for BMAP28(1–18). Thus, the above results clearly demonstrate that the sensitivity of hemolysis was similar, but the threshold peptide concentration required for 50% hemolysis was lower for P1m indicating an amplifying effect with respect to both P1 and BMAP28(1–18). Thus, P1m demonstrated the maximum hemolytic activity.

In summary, a single residue mutation identified by MD-guided rational design enhanced the membrane activity of the peptide against all three types of membranes. The Hill parameters suggest a possible mechanism of membrane activity against these peptides. A high value of Hill coefficient (*n*) indicates aggregation of peptides on the membrane leading to a sensitive response, while a lower half saturation constant indicates higher potency demonstrating amplification. Our study suggests that for a Gram-positive membrane the aggregation of peptides was the key factor deciding the antibacterial activity. Since in this case, P1m demonstrated the highest sensitivity with a high value of *n* with marginal variation in *K*-values. Whereas in the case of Gram-negative membrane, both aggregation (higher *n*-values) and higher binding affinity (lower *K*-values) influence the potency. In case of RBCs, all the three peptides demonstrated similar aggregation (with low *n*-values), however with significantly different binding affinity (varying *K*-values). P1m displayed the highest binding affinity while P1 displayed the lowest. These studies clearly demonstrated that a single residue change in the peptide sequence resulted in altering the response behavior by perturbing both the aggregation and the binding affinity. Further, these effects manifested differently in the three types of membranes, with P1m demonstrating higher aggregation in Gram-positive membrane, increasing both aggregation and binding in case of Gram-negative and increasing only the binding in case of RBCs. The higher *K*-values of all the peptides for RBCs as compared to the bacterial systems suggest that the peptides exhibit more potent antibacterial as compared to hemolytic activity.

## Conclusions

This study demonstrates that prediction algorithms along with MD simulations can be exploited for design of novel AMPs. The mutant peptide (P1m) demonstrated enhanced membrane activity as compared to the wild-type peptide (P1) against all three types of membranes studied viz., Gram-positive, Gram-negative and human erythrocytes. Helical content is known to influence membrane activity and CD studies revealed that P1m exhibited higher helical content as compared to P1 (Figure 3). Another interesting outcome arising from the study of death rate constant of *S. aureus* is the drastic change in the behavior of P1m owing to a single residue mutation. This concentration dependent transition from monophasic to biphasic behavior illustrates the effect of minor sequence variations on peptide behavior resulting in altered activity. A worthwhile observation was that the effect of the same mutation varies with the type of membrane. For e.g., in case of Gram-positive bacteria, the sensitivity of the response is much more as compared to Gram-negative bacteria; while for erythrocytes the amplification dramatically increases. These response behaviors can be hypothesized to be linked with factors associated with microbial killing such as aggregation and membrane binding strength of the peptides (Frecer et al., 2004). Thus, the potency of the peptide can be correlated to both amplification and sensitivity and these are important factors in the design of antimicrobial peptides.

## Ethics statement

This study was carried out in accordance with the recommendations of NIRRH Human Ethics committee with written informed consent from all subjects. All subjects gave written informed consent in accordance with the Declaration of Helsinki. The protocol was approved by the NIRRH Human Ethics committee [Project No: 296/2016].

## Author contributions

SI-T conceived the study. FW and SJ performed the experiments. FW, SG, and EM performed MD simulation and its analysis. FW, SJ, EM, KV, and SI-T wrote the paper. KV did the killing kinetics data interpretation and analysis. TM helped with the experimental assay. All authors have read and approved the manuscript.

### Conflict of interest statement

The authors declare that the research was conducted in the absence of any commercial or financial relationships that could be construed as a potential conflict of interest.
