# Supplementary material for: Designing Antibacterial Peptides with Enhanced Killing Kinetics
Source: Front Microbiol. 2018 Feb 23;9:325. doi: 10.3389/fmicb.2018.00325 (PMC5829097; doi:10.3389/fmicb.2018.00325)
Supplement: Supplementary file 8 [file Table1.docx]

**Supplementary Table 1**: Peptide-lipid system generated using CHARMM-GUI

| System | Lipid molecules | Water model | Force field |
| --- | --- | --- | --- |
| P1, P1m, P1m1 and P1m2 - SDS micelle | 60 | SPC | CHARMM27 |
| P1 & P1m - DPC micelle | 60 | TIP3P | CHARMM36 |
| BMAP28(1-18) - POPC:POPG (2:1) bilayer | 84(POPC) 42(POPG) | TIP3P | CHARMM36 |
