# Supplementary material for: Designing Antibacterial Peptides with Enhanced Killing Kinetics
Source: Front Microbiol. 2018 Feb 23;9:325. doi: 10.3389/fmicb.2018.00325 (PMC5829097; doi:10.3389/fmicb.2018.00325)
Supplement: Supplementary file 9 [file Table2.docx]

**Supplementary Table 2:** Calculated eccentricity and ratios of moment of inertia of SDS and DPC micelle in the presence and absence of peptides

| Peptides | e | | Ratios of MOI (R1:R2:R3) | |
| --- | --- | --- | --- | --- |
|  | **SDS** | **DPC** | **SDS** | **DPC** |
| No peptide | 0.02 | 0.05 | 1.01:1.06:1 | 1.02:1.14:1 |
| P1 | 0.12 | 0.10 | 1.16:1.27:1 | 1.13:1.23:1 |
| P1m1 | 0.13 | - | 1.18:1.29:1 | - |
| P1m2 | 0.14 | - | 1.19:1.31:1 | - |
| P1m | 0.14 | 0.11 | 1.20:1.32:1 | 1.15:1.25:1 |
