# Supplementary material for: Designing Antibacterial Peptides with Enhanced Killing Kinetics
Source: Front Microbiol. 2018 Feb 23;9:325. doi: 10.3389/fmicb.2018.00325 (PMC5829097; doi:10.3389/fmicb.2018.00325)
Supplement: Supplementary file 10 [file Table3.docx]

**Supplementary Table 3:** Constituents of relative free binding energy of P1, P1m, P1m1 and P1m2 with SDS in kJ/mol

| Peptides | van der Waal energy | Electrostatic energy | Polar solvation energy | SASA energy | Binding Energy |
| --- | --- | --- | --- | --- | --- |
| P1 | -563.5+/- 48.1 | -10586.6+/- 361.7 | 1547.4+/- 207.7 | -66.6+/- 4.7 | -9669.3+/- 260.3 |
| P1m1 | -527.9+/-32.6 | -10583.2 +/- 357.2 | 1514.4 +/- 161.4 | -64.8 +/- 4.0 | -9661.5 +/- 288.2 |
| P1m2 | -562.9 +/- 39.3 | -10677.2 +/- 266.5 | 1606.1 +/- 158.4 | -67.7 +/- 3.7 | -9701.7 +/- 182.3 |
| P1m | -555.7+/- 41.8 | -10764.9+/- 298.3 | 1575.6+/- 189.6 | -67.3+/- 3.8 | -9812.3+/- 217.3 |
