# Supplementary material for: Designing Antibacterial Peptides with Enhanced Killing Kinetics
Source: Front Microbiol. 2018 Feb 23;9:325. doi: 10.3389/fmicb.2018.00325 (PMC5829097; doi:10.3389/fmicb.2018.00325)
Supplement: Supplementary file 11 [file Table4.docx]

**Supplementary Table 4:** Constituents of relative free binding energy of P1 and P1m with DPC in kJ/mol

| Peptides | van der Waal energy | Electrostatic energy | Polar solvation energy | SASA energy | Binding Energy |
| --- | --- | --- | --- | --- | --- |
| P1 | -489.63 +/- 44.82 | -1085.78 +/- 237.22 | 1055.55 +/- 210.11 | -68.49 +/- 5.02 | -588.35 +/- 110.59 |
| P1m | -465.21+/- 43.20 | -1170.37 +/- 192.21 | 1101.41 +/- 186.56 | -65.97 +/- 5.12 | -600.14 +/- 97.25 |
