# Supplementary material for: Designing Antibacterial Peptides with Enhanced Killing Kinetics
Source: Front Microbiol. 2018 Feb 23;9:325. doi: 10.3389/fmicb.2018.00325 (PMC5829097; doi:10.3389/fmicb.2018.00325)
Supplement: Supplementary file 13 [file DataSheet1.DOCX]

Supplementary Material

Designing antibacterial peptides with enhanced killing kinetics

**Faiza Hanif Waghu, Shaini Joseph, Sanket Ghawali, Elvis A. Martis, Taruna Madan, Venkatesh V. Kareenhalli and Susan Idicula-Thomas^*^**

*** Correspondence:** Susan Idicula-Thomas: thomass@nirrh.res.in

# Supplementary Data

# Hill equation fit

# *Quantification of antimicrobial activity*

# The % death value after 18h incubation of the organisms (E. coli ATCC 8739 and S. aureus ATCC 25923) with the peptides was fitted using the Hill equation:

# %Death= $\mathbf{100*}\left[ \frac{\mathbf{C}^{\mathbf{n}}}{\mathbf{K}^{\mathbf{n}}\mathbf{+}\mathbf{C}^{\mathbf{n}}} \right]$

# The data was fitted to the above equation to yield n and K values and are represented in Table 4 in the main text.

# *Cytotoxicity assay against normal human erythrocytes*

# The % hemolysis was fitted using Hill equation to obtain n and K values. These values are also referred in Table 4 in the main text.

### Study of death rate of *S. aureus* ATCC 25923

To further study the effect of mutation on behaviour of peptides, the first order rate constant for the death of *S. aureus* ATCC 25923 was determined by performing time-kill assays using BMAP28(1-18), P1 and P1m. The following equations were obtained by the fit.

For BMAP28(1-18),

$\frac{K}{27}=(\frac{C^{2.4}}{{3.1}^{2.4}+C^{2.4}})(1+7.6*{10}^{-4}e^{0.15C})$ (1)

For P1,

$\frac{K}{28}=(\frac{C^{2.3}}{{8.4}^{2.3}+C^{2.3}})$ (2)

For P1m,

$\frac{K}{30}=(\frac{C^{2.1}}{{9.65}^{2.1}+C^{2.1}})(1+7*{10}^{-5}e^{0.19C})$ (3)

In the first phase, the rate constant saturated to about 28.5 h^-1^ up to a peptide concentration of 30µM. Beyond this peptide concentration, the rate constant exponentially increased resulting in rapid killing of cells. For both P1m and BMAP28(1-18), the death rate constant of about 60 h^-1^ was observed at 50µM of the peptide indicating that only about 40 s is sufficient to reduce the cell number by half. Although P1m and BMAP28(1-18) demonstrated similar killing behaviour, higher amount of P1m was needed as compared to BMAP28(1-18) for the initial saturation. The behaviour of the rate constant was captured as a product of two functions, namely, an initial region represented by Hills Equation indicating a saturating behaviour, and exponential increase post saturation (Equation 1-3). The Hills constant for the two peptides are similar with a value of 2.1 and 2.4 for P1m and BMAP28(1-18), respectively, whereas the half saturation constant is lower for BMAP28(1-18) as compared to P1m (3.1 and 9.65µM). A Hills coefficient greater than 2 indicates a sensitive response requiring only about less than 9 fold change in the concentration to reach saturation. But a lower half saturation constant for BMAP28(1-18) indicates higher affinity of binding of the peptides to the cell membrane for BMAP28(1-18) compared to that for P1m. Thus, BMAP28(1-18) shows a higher affinity at lower concentrations as compared to that of P1m. but has a similar exponential increase in the rate constant beyond saturation (i.e. for greater than 30µM of peptide concentration). An interesting observation was noted for the case of P1, wherein, the initial saturation of the rate constant was similar to that of P1m, that is it has a similar Hills coefficient (2.3) and a half saturation constant (of 8.4µM) as seen in Equation 2. This indicates that the affinity and sensitivity is similar for P1 and P1m in the initial saturation phase (for less than 30µM). However, beyond the saturation concentration, P1 did not show an increase in the rate constant as observed for both BMAP28(1-18) and P1m in the range of peptide concentration studied. This dramatic effect was caused due to a single change in the residue of an amino-acid. It may be possible that the second phase for P1 may occur at a higher concentration of peptide.

**Evaluating Hill Equation parameters:**

The percentage death was fitted to a sigmoidal input-output relationship through Hill's Equation as given below.

$$Y= \frac{X^{n}}{X^{n}+K^{n}}$$

Here, Y is the normalized percentage death taking values in the range [0,1] and X is the concentration of the antimicrobial peptide. The data for value of Y was obtained through experiments. It can be noted that the Hill's Equation has two parameters namely, n and K, Hill's Coefficient and Half Saturation Constant, also referred to as K_0.5_. The values of the parameters are specific to a system. The Hill Equation can be linearized as follows.

The linear equation was fitted by plotting LHS of the above equation versus *ln(X)*. The slope of the best fit line equation (linear regression) will yield the value of Hill's coefficient and the value of the intercept can be further used to evaluate the value of the half saturation constant, K. The Hill coefficient (n) determines the sensitivity of the response (steepness of the sigmoidal curve) and the half saturation constant (K) determines the threshold input needed for the response (equivalent to EC_50_ value).

**Evaluating kinetic rate constant:**

The initial rate constant was determined by fitting the first order death kinetics. The CFU (C) of the organism/cell was determined experimentally in time. The first order rate constant, k, was evaluated by linear fitting the equation given below:

ln(C/C_0_) = kt

# Supplementary Figures and Tables

## Supplementary Figures

##
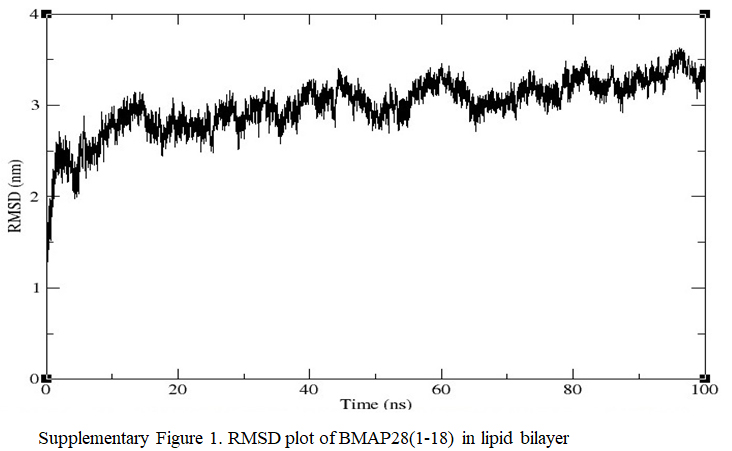


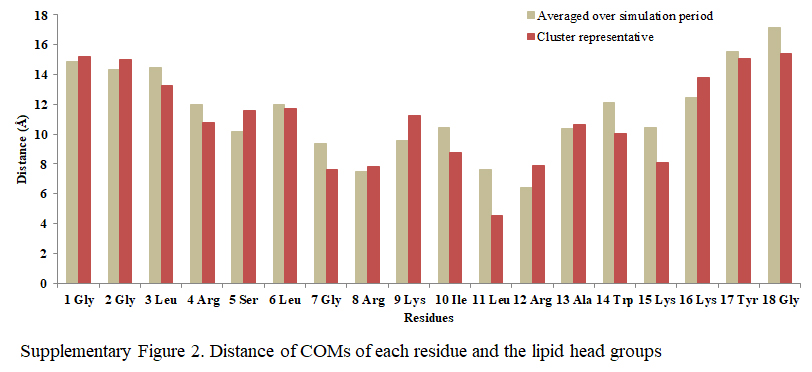


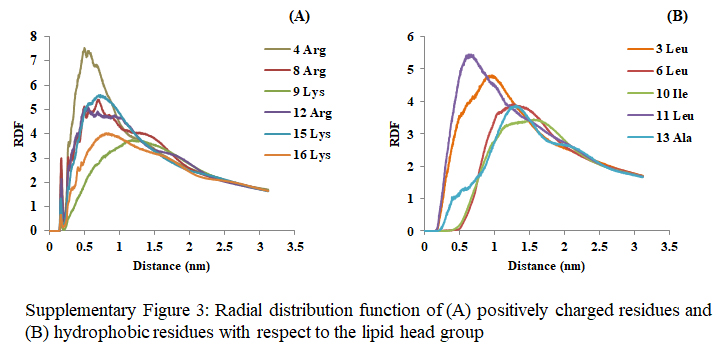


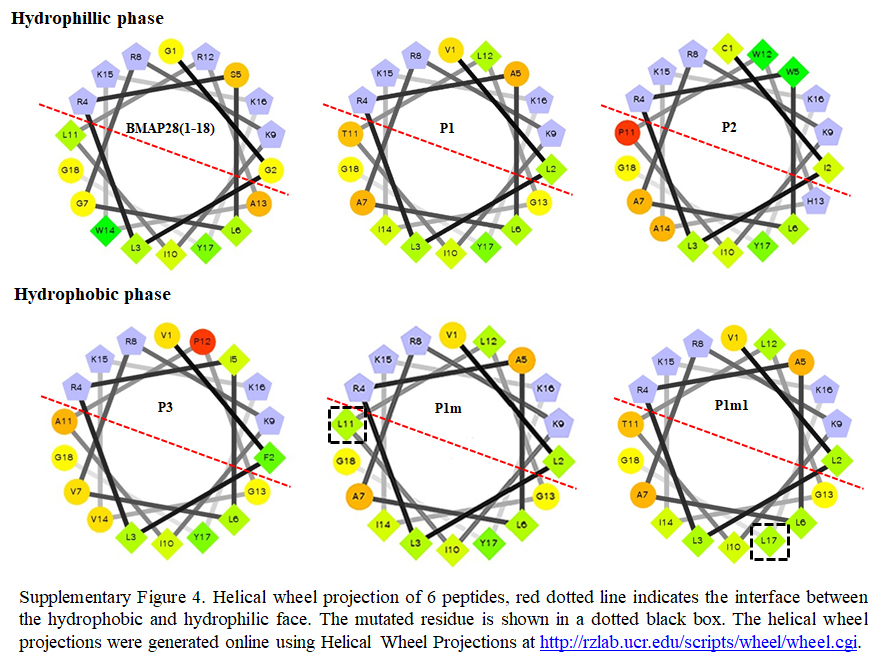


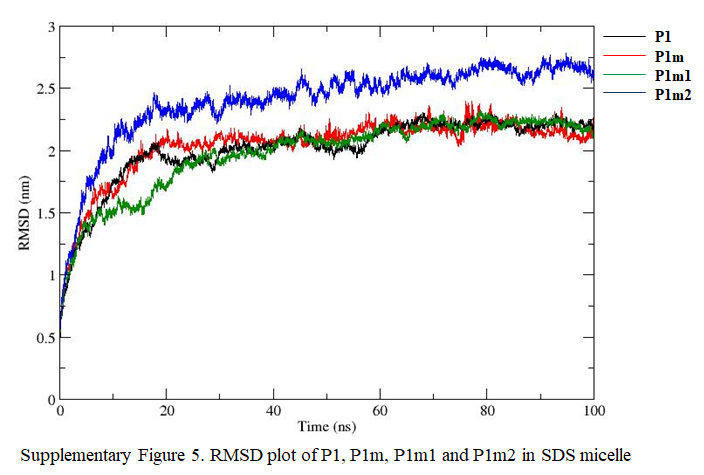


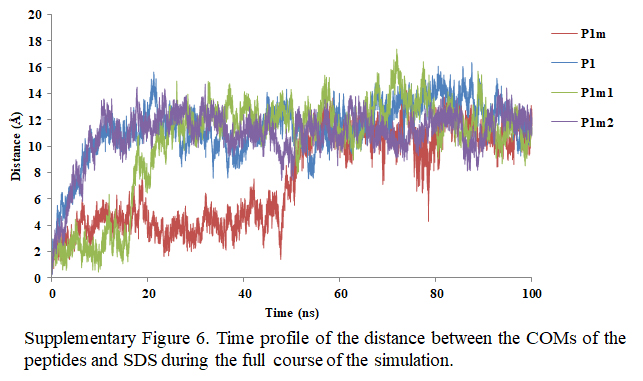


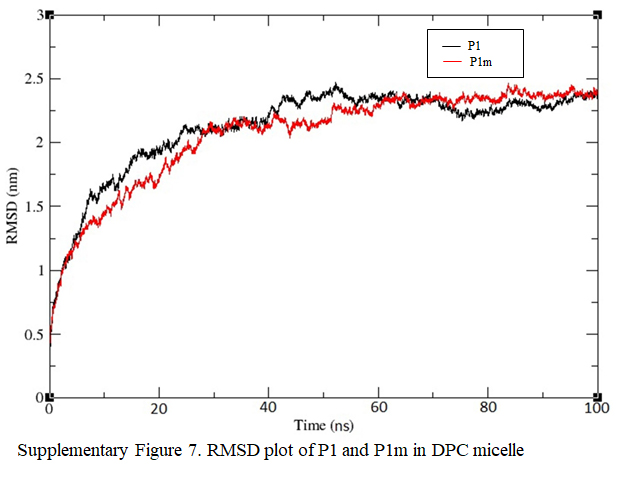


## Supplementary Tables

**Supplementary Table 1**: Peptide-lipid system generated using CHARMM-GUI

| System | Lipid molecules | Water model | Force field |
| --- | --- | --- | --- |
| P1, P1m, P1m1 and P1m2 - SDS micelle | 60 | SPC | CHARMM27 |
| P1 & P1m - DPC micelle | 60 | TIP3P | CHARMM36 |
| BMAP28(1-18) - POPC:POPG (2:1) bilayer | 84(POPC) 42(POPG) | TIP3P | CHARMM36 |

**Supplementary Table 2:** Calculated eccentricity and ratios of moment of inertia of SDS and DPC micelle in the presence and absence of peptides

| Peptides | e | | Ratios of MOI (R1:R2:R3) | |
| --- | --- | --- | --- | --- |
|  | **SDS** | **DPC** | **SDS** | **DPC** |
| No peptide | 0.02 | 0.05 | 1.01:1.06:1 | 1.02:1.14:1 |
| P1 | 0.12 | 0.10 | 1.16:1.27:1 | 1.13:1.23:1 |
| P1m1 | 0.13 | - | 1.18:1.29:1 | - |
| P1m2 | 0.14 | - | 1.19:1.31:1 | - |
| P1m | 0.14 | 0.11 | 1.20:1.32:1 | 1.15:1.25:1 |

**Supplementary Table 3:** Constituents of relative free binding of P1 and P1m with SDS in kJ/mol

| Peptides | van der Waal energy | Electrostatic energy | Polar solvation energy | SASA energy | Binding Energy |
| --- | --- | --- | --- | --- | --- |
| P1 | -563.5+/- 48.1 | -10586.6+/- 361.7 | 1547.4+/- 207.7 | -66.6+/- 4.7 | -9669.3+/- 260.3 |
| P1m1 | -527.9+/-32.6 | -10583.2 +/- 357.2 | 1514.4 +/- 161.4 | -64.8 +/- 4.0 | -9661.5 +/- 288.2 |
| P1m2 | -562.9 +/- 39.3 | -10677.2 +/- 266.5 | 1606.1 +/- 158.4 | -67.7 +/- 3.7 | -9701.7 +/- 182.3 |
| P1m | -555.7+/- 41.8 | -10764.9+/- 298.3 | 1575.6+/- 189.6 | -67.3+/- 3.8 | -9812.3+/- 217.3 |

**Supplementary Table 4:** Constituents of relative free binding of P1 and P1m with DPC in kJ/mol

| Peptides | van der Waal energy | Electrostatic energy | Polar solvation energy | SASA energy | Binding Energy |
| --- | --- | --- | --- | --- | --- |
| P1 | -489.6 +/- 44.8 | -1085.8 +/- 237.2 | 1055.6 +/- 210.1 | -68.5 +/- 5.0 | -588.3 +/- 110.6 |
| P1m | -465.2+/- 43.2 | -1170.4 +/- 192.2 | 1101.4 +/- 186.6 | -65.9 +/- 5.1 | -600.1 +/- 97.3 |
